# What is new in cancer-associated fibroblast biomarkers?

**DOI:** 10.1186/s12964-023-01125-0

**Published:** 2023-05-04

**Authors:** Zehua Zhao, Tianming Li, Yuan Yuan, Yanmei Zhu

**Affiliations:** 1 Department of Pathology, Affiliated Cancer Hospital of Dalian University of Technology (Liaoning Cancer Hospital and Institute, Cancer Hospital of China Medical University), No. 44 of Xiaoheyan Road, Dadong District, Shenyang, 110042 China; 2 Tumor Etiology and Screening Department of Cancer Institute and General Surgery, The First Hospital of China Medical University, Shenyang, China; 3 Key Laboratory of Cancer Etiology and Prevention in Liaoning Education Department, The First Hospital of China Medical University, Shenyang, China; 4 Key Laboratory of GI Cancer Etiology and Prevention in Liaoning Province, The First Hospital of China Medical University, No. 155 of Nanjing Road, Heping District, Shenyang, 110001 China

**Keywords:** Cancer-associated fibroblasts, Biomarker, Heterogeneity, Targeted therapy

## Abstract

The tumor microenvironment is one of the important drivers of tumor development. Cancer-associated fibroblasts (CAFs) are a major component of the tumor stroma and actively participate in tumor development, invasion, metastasis, drug resistance, and other biological behaviors. CAFs are a highly heterogeneous group of cells, a reflection of the diversity of their origin, biomarkers, and functions. The diversity of CAF origin determines the complexity of CAF biomarkers, and CAF subpopulations expressing different biomarkers may play contrasting roles in tumor progression. In this review, we provide an overview of these emerging CAF biomarkers and the biological functions that they suggest, which may give a better understanding of the relationship between CAFs and tumor cells and be of great significance for breakthroughs in precision targeted therapy for tumors.

Video Abstract

**Supplementary Information:**

The online version contains supplementary material available at 10.1186/s12964-023-01125-0.

Tumor tissue comprises tumor cells and the extracellular stroma. Since Paget described the importance of the surrounding environment to cancer development as the “seed and soil” theory in 1889, the role of the tumor microenvironment (TME) in understanding tumorigenesis and progression has become more important. The TME refers to the interstitial environment around tumor cells, which includes not only the extracellular matrix (ECM), blood vessels, and factors such as cytokines and growth factors that affect tumor development, but also many kinds of cells, such as fibroblasts, immune cells, endothelial cells, and adipocytes. Accordingly, the TME is closely related to the diverse biological behaviors of tumors [1–3].

Cancer-associated fibroblasts (CAFs) are an important component of the tumor stroma. CAFs interact with tumor cells in various ways, such as by remodeling the ECM and secreting cytokines and exosomes and via metabolic reprogramming, and actively participate in a variety of biological behaviors, including tumor development, invasion, metastasis, and drug resistance (Fig. 1). Recent studies have found that the role of CAFs in tumors is bidirectional. CAFs can facilitate tumor development through metabolic interactions, thereby promoting angiogenesis and immunosuppression; CAFs can also effectively impair tumor invasiveness and increase drug sensitivity and be positively associated with a better prognosis, indicating their tumor-suppressive effects.

**Fig. 1:**
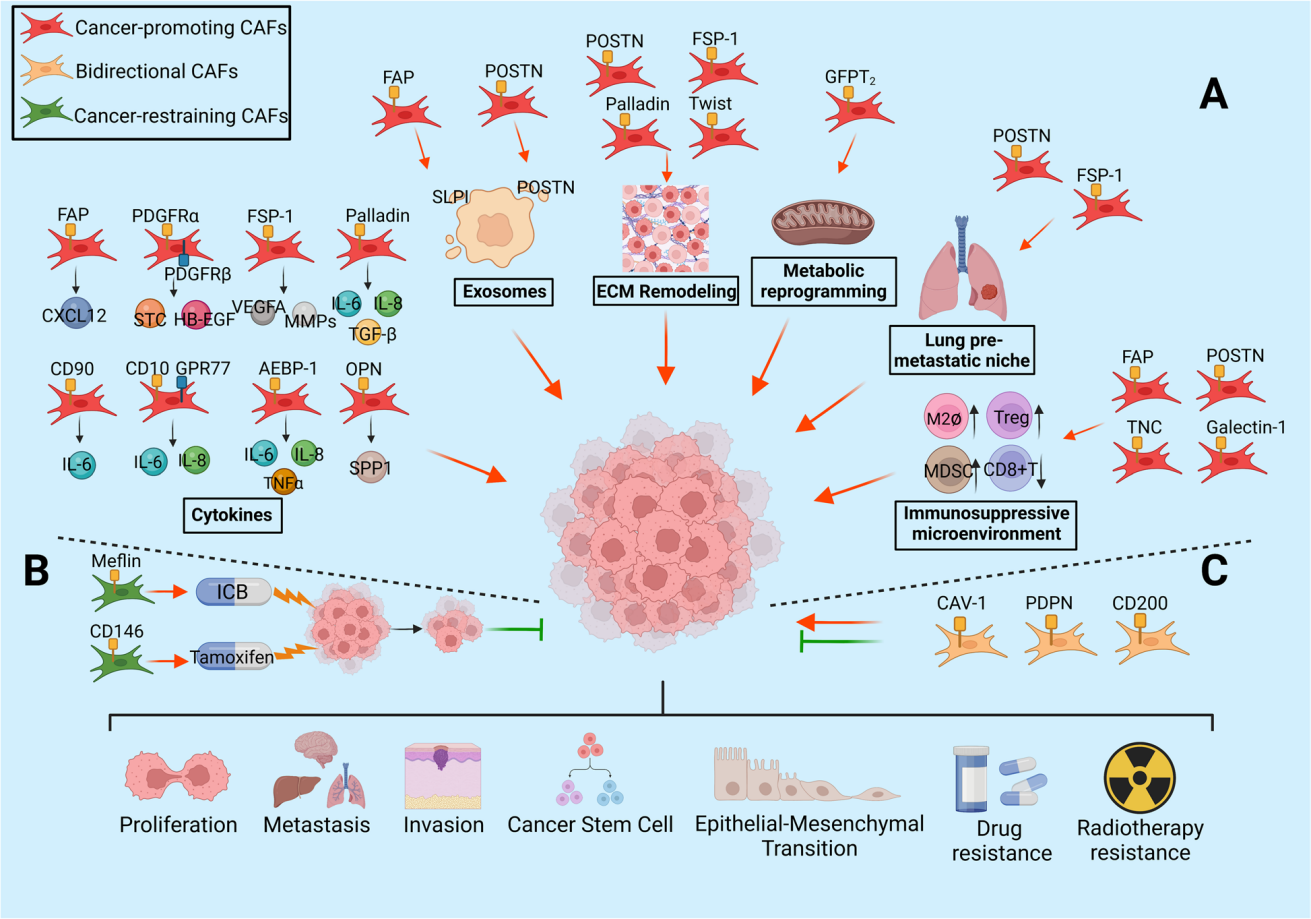
CAF biomarkers and functions. CAF subgroups identified by different biomarkers play distinct roles in tumor microenvironment. **A** Biomarkers suggesting cancer-promoting functions. Most biomarkers represent cancer-promoting CAFs including traditional CAF biomarkers (FAP, POSTN, PDGFRα/β, and FSP-1) as well as some emerging markers (CD90, Palladin, OPN, AEBP1, Twist, TNC, Gallectin1, CD10 and GPR77). They promote cancer progression though secreting cytokines and exosomes, remolding ECM, metabolic reprogramming, forming anterior niche in lung metastasis and immunosuppressive microenvironment, etc. **B** Biomarkers indicating cancer-restraining functions. Meflin^+^ CAFs are associated with favorable therapeutic response to ICB; CD146^+^CAF maintains sensitivity to tamoxifen of ER positive breast cancer. Both are related to better pathological histological features and prognosis of patients. **C** Biomarkers showing bidirectional functions. Cav-1, PDPN and CD200 labeled CAFs have both tumor-promoting and tumor-suppressing functions

The reason why CAFs have such a complex function is that CAFs are a highly heterogeneous group of cells, which is mainly due to their diverse origins. CAFs can be derived from a variety of cells, including normal fibroblasts (NFs) in the peritumoral stroma, bone marrow-derived mesenchymal stem cells (MSCs), stellate cells, epithelial cells, and endothelial cells. Other precursors of CAFs include vascular smooth muscle cells, perivascular cells, adipocytes, and adipose stem cells [4]. CAFs of different cell sources can express different biomarkers, and different markers can reflect the different biological properties of CAFs. The diversity of CAF cell origins determines the complexity of CAF markers. In recent years, a multitude of clinical observations have indicated that CAF subgroups expressing different markers play distinct roles in the tumor development process of various cancers, such as cancer-promoting (Table 1), cancer-restraining (Table 2), and bidirectional function (Table 3). Therefore, the study of CAF markers is of great importance for achieving breakthroughs in precision targeted therapy. In recent years, with the emphasis on tumor immune microenvironment and the development of molecular techniques, such as single-cell sequencing, there has been a great progress in the study of CAF markers. In this review, we incorporate some new CAF markers including Meflin and CD200, and also delve into new mechanisms of the so-called “old” CAF markers acting on tumor cells, such as leading to immunosuppressive TME status. We will discuss all these markers by origin and function, which will enable us to better understand the role of CAF markers in tumorigenesis and tumor-targeted therapy.

**Table 1:** The role of CAFs labeled by cancer-promoting markers in various kinds of cancer

**Bio** **marker**	**Main** **origin**	**Description**	**Roles in cancers**			
			**Pancreatic cancer**	**Breast cancer**	**Colorectal cancer**	**Lung cancer**
FAP	NFs	Type II intact membrane protein of serine protease family, including two subunits, α and β	Regulating accumulation of regulatory T cells, inhibition T cell activity, secreting CXCL12 and marginalized T cells through CXCL12-CXCR4 signaling, leading to tumor immunosuppression; Tumor promoting	Enriched in HER2-positive and triple-negative breast cancer stroma; expressing PD-1/2, which can bind to PD-1 expressed by T cells and inhibiting T cell activity	Interaction of FAP^+^ CAFs and SPP1^+^ macrophages contributing to desmoplastic tumor microenvironment and correlating with immunotherapy resistance	Correlating with the immunosuppressive TME status; predictive biomarker of resistance to PD-1 blockade; poor prognosis marker
α-SMA		Cytoskeleton protein	α-SMA^high^FAP^high^ -myCAF α-SMA^low^FAP^low^ -iCAF; relationship with survival is contradictory; inhibition IL-6 from α-SMA^+^CAF improving gemcitabine efficacy	Improving tumor growth via secretion of osteopontin; poor prognosis	Poor survival rates	MYH11^+^ α-SMA^+^ CAF and FAP^+^ α-SMA^+^ CAF driving T cell marginalization
POSTN		Stromal cell protein	Associated with highly cellular tumors and macrophage infiltrates and shorter overall survival	Playing an important role in carcinogenesis in situ and perhaps beyond for those cancers that become invasive	Correlated with tumor progression, lymph node and distant metastases, and poor clinical outcomes	Prognostic marker
PDGFRα/β		Tyrosine kinase receptor	Lymphatic invasion and lymph node metastasis; poor prognosis	PDGFRβ interacting with integrin A11 and leading to more aggressive behavior by activating JNK signaling	PDGFRβ^+^CAFs increasing the invasion and metastasis ability in a secreted glycoprotein stanniocalcin-1 dependent manner	Promoting recruitment of fibroblasts to the tumor infiltration front
FSP-1		Small calcium binding protein	Identifying a unique population of fibroblasts with minimal overlap with markers for αSMA and PDGFRβ	Positive in primary breast cancer and in matched LNMs; inducing metastasis; associated with poor patient survival	Associated with poor patient survival	Lung premetastasis niche formation
Palladin		Actin binding protein	Secreting immunosuppressive cytokines to maintain the characteristics of iCAFs; producing functional desmoplastic ECMs to support cancer cell survival and proliferation; facilitating the invasion of cancer cells by remodeling ECM though regulating the small GTPase Cdc42; independent poor prognostic factor	As Akt1-specific substrate and regulating breast cancer cell migration	Correlated with reduced survival and relapse	Affecting the behavior of CAFs, leading to a pro-invasive henotype
Twist1		A basic helix-loop-helix transcription factor	-	Tumor promoting; related to shorter survival	Promoting matrix stiffness	-
GFPT2		Glutamine-Fructose-6-Phosphate Transaminase 2	-	-	Regulating immunosuppression through JAK/STAT signaling pathway; associated with poorer pathological characteristics and OS	Regulating metabolic reprogramming and negatively correlates with prognosis
Vim	MSCs	Cytoskeletal protein	Poor survival; pro-tumoural profile	Associated with indices of poor prognosis	Independent prognostic factor for recurrence; related to shorter survival and chemoresistance	Promoting lung cancer metastasis by surrounding the cancer cells sprouting from primary tumor
TNC		Extracellular matrix protein	Perineural invasion, high loco-regional recurrence, proliferation and invasion; poor prognosis	Related to LN metastasis and poorer outcome	Promoting EMT and proliferation; involved in tumor growth and metastasis via Hedgehog signaling; poor prognosis	Poorer clinical outcome biomarker
CD90		GPI-anchored glycoprotein	Promoting pancreatic cancer development	-	Supporting the stemness of tumor cells and inducing an immune adaptive inflammatory response	Promoting tumor cell invasion; poor prognosis
CD10		Metalloendoprotease	-	Supplying paracrine IL-8 and IL-6 through NF-κB signaling, forming a niche that protects CSC,leading to chemotherapy resistance and poor survival	-	Same to breast cancer
GPR77		Non-G protein coupled receptor	-	-	-	
Galectin 1	Stellate cells	β-galactoside binding protein	Tumor cell proliferation, angiogenesis, invasion, metastasis and inflammation	Immunosuppressive microenvironment; associated with high tumor grade and lymph node involvement; poor prognosis	Promoting CIC features and disease dissemination	Poorer clinical outcome biomarker
AEBP1	Epithelial cells	Multifunctional protein	-	-	Promoting proliferation, invasion, migration and metastasis by activating NF-κB signaling	An independent poor prognostic factor
OPN	Not clear	secretory phosphoprotein	Promoting cancer stemness via SPP1-CD44 axis	Poor prognosis	Contributing to tumorigenesis by activation of the STAT3/PPARg pathway;poor prognosis	Promoting invasiveness and proliferation;poor prognosis

**Table 2:** The role of CAFs labeled by cancer-restraining markers in various kinds of cancer

**Bio** **marker**	**Main** **origin**	**Description**	**Roles in cancers**			
			**Pancreatic cancer**	**Breast cancer**	**Colorectal cancer**	**Lung cancer**
Meflin	Not clear	Glycosylated phosphatidylinositol anchored protein	Attenuating tumor aggressiveness; positively correlated with higher differentiated pathological histological features and better prognosis	-	-	Sensitive to ICB treatment
CD146	Vascular endothelial cells	Cell adhesion molecule	Related to low histological grade and high-grade pancreatic intraepithelial neoplasia; inhibiting invasion and migration of cancer cells; better prognosis marker	Maintaining ER expression, estrogen-dependent proliferation and sensitivity to tamoxifen	-	-

**Table 3:** The role of CAFs labeled by bidirectional function markers in various kinds of cancer

**Bio** **marker**	**Main** **origin**	**Description**	**Roles in cancers**			
			**Pancreatic cancer**	**Breast cancer**	**Colorectal cancer**	**Lung cancer**
Cav-1	NFs	A scaffold protein	Higher CA19-9 level and rates of advanced tumor stage; lower DFS and OS	Cav-1 deficiency is associated with early recurrence, late tumor stage, lymph node metastasis, triamcinolone resistance and poor prognosis; Cav-1 upregulation hardening TME to promote tumor cell invasion and metastasis	Tumor-promoting	A marker of good efficacy for nab-paclitaxel treatment
PDPN	Vascular endothelial cells	Mucin-type salivary glycoprotein	Associated with immune-related signatures and recruitment of dendritic cells; enhancing the progression, and serving as an independent predictor of poor outcome	Good independent prognostic marker	PNPD^high^ CAFs are protective against cell invasion; associated with favorable clinicopathological parameters and prolonged DFS	PDPN^+^CAFs are related to immunosuppressive microenvironment; leading to primary resistance to EGFR-TKI by activating MAPK signaling; a risk factor for recurrence
CD200	Not clear	OX2 membrane glycoprotein	No clear correlation with PFS and OS	-	-	Increasing sensitivity of EGFR mutation lung cancer to the EGFR-TKI

## CAF markers with cancer promotion

### Markers of CAFs derived from NFs

#### Fibroblast activation protein

Fibroblast activation protein (FAP) is a type II membrane protein of the membrane-binding serine protease family comprising α and β subunits. Although it normally exists as a homodimer, when activated, it assembles into a heterodimer composed of the α and β subunits and participates in the migration of fibroblasts to the collagen matrix. FAP is highly expressed in several cancerous myofibroblasts and is considered an important marker of CAFs [5]. FAP^+^ CAFs mainly originate from NFs [6].

FAP promotes the progression of various cancers. It boosts the metastasis of phyllodes tumor [7], the invasive behavior of bladder high-grade urothelial carcinoma (UC) [8], prostate cancer cell invasion and proliferation [9], and cancer development by epithelial-mesenchymal transition (EMT) via the Wnt/β-catenin signaling pathway in gastric cancer [10]. FAP correlates with adverse clinicopathological factors, such as invasion depth in esophageal adenocarcinoma (EAC) [11], large tumor diameter (> 7 cm), advanced stage (pT3/4), high grade (G3/4), sarcomatoid transformation, tumor necrosis, and early lymph node metastasis of clear cell renal cell carcinoma (CCRCC) [12]. In ovarian cancer, secretory leukocyte protease inhibitor (SLPI) protein derived from FAP^high^ α-SMA^low^ CAFs can be encapsulated in extracellular vesicles (EVs) and delivered to cancer cells, promoting cell proliferation, adhesion, invasion, and migration via the PI3K/AKT signaling pathway [13]. It is associated with poor prognosis of NSCLC [14], gastric cancer [10], CCRCC [12], ovarian cancer [13], and bladder high-grade UC [8], but not cervical cancer [15]. FAP^+^ CAFs are independent predictive factors to worse tumor regression grade (TRG), that means chemoresistance in gastric cancer [16].

FAP is correlated with immunosuppressive TME status and leads to immunotherapy resistance. The reciprocity of FAP^+^ fibroblasts and SPP1^+^ macrophages has been revealed through single-cell and spatial analysis in colorectal cancer (CRC). The reciprocity might be adjusted by TGF-β, IL-1, and chemerin, correlate with desmoplastic TME, and lead to immunotherapy resistance [17]. In gastric cancer, FAP expression is related to immunosuppressive cell infiltration, such as that of M2 macrophages (CD163^+^) and myeloid-derived suppressor cells (MDSCs) (CD11b^+^/CD33^+^). In advanced non-small cell lung cancer (NSCLC), FAP is related to a reduced density of CD8^+^ T cells and immunosuppressive TME status [18, 19]. In HER2^+^ and triple-negative breast cancer, the CAF subpopulation with high FAP expresses programmed death ligand-1/2 (PD-L1/2), which can bind to PD-1 expressed by T cells and directly inhibit T cell activity [20]. In a mouse model of pancreatic cancer, FAP^+^ CAFs are the main source of CXCL12 that combines with CXCR4 on the surface of cancer cells and marginalized T cells through a CXCL12–CXCR4 signaling mechanism, leading to tumor immunosuppression [21]. FAP also promotes the growth of intrahepatic cholangiocarcinoma cells by attracting MDSCs through CCL2 [22]. In summary, FAPs can be used as a biomarker to predict immunosuppressant resistance [14].

All of the above indicates that FAP^+^ CAFs are potential therapeutic targets for solid tumors. The vast majority of therapeutic approaches targeting FAP^+^ CAFs involve their depletion. The strategies include FAP antibodies [23–25], genetic deletion [26], pharmacological inhibition (PT630, PT-100) [27, 28], conditional ablation using diphtheria toxin [21] or FAP-PE38 [25], and novel FAP-targeting immunotherapies such as DNA vaccination [29] and chimeric antigen receptor (CAR) T cells [30, 31].

#### α-Smooth muscle actin

α-Smooth muscle actin (α-SMA) belongs to the actin family of cytoskeletal proteins and is widely known for its role in wound healing. α-SMA is frequently expressed in CAFs and is one of the preferred markers for identifying CAFs [32, 33]. α-SMA^+^ CAFs are mainly derived from NFs. Other sources include stellate cells, epithelial cells, vascular endothelial cells, vascular smooth muscle cells, and MSCs [4, 6, 33, 34].α-SMA^+^ CAFs participate in cancer progression. α-SMA-expressing CAFs can enhance the colony formation, proliferation, and invasiveness of pancreatic cancer cells [35]. α-SMA^+^ CAFs also promote the proliferation of bile duct epithelial cells and trigger their entry into the S + G2/M phase, resulting in tumor promotion of cholangiocarcinoma [36]. α-SMA^+^ CAFs secreting OPN promote luminal breast cancer growth [37]. However, studies of a pancreatic ductal adenocarcinoma (PDAC) mouse model have also identified tumor-restraining α-SMA^+^ CAFs (rCAFs) and tumor-promoting FAP^+^ CAFs (pCAFs), which are involved in the aggregation of regulatory T cells [38], suggesting the complexity of α-SMA^+^ CAF function.

**Fig. 2:**
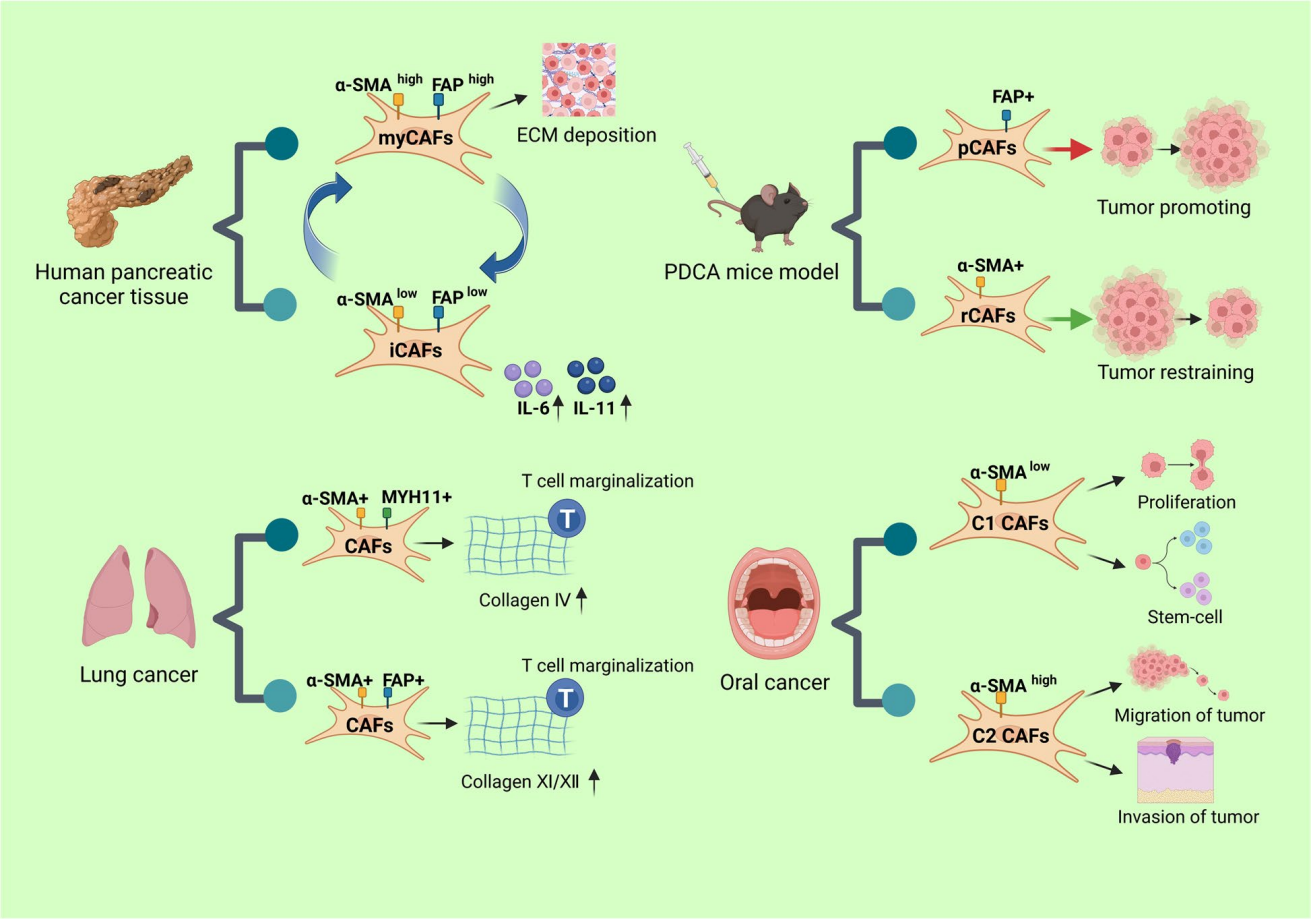
Subgroups of CAFs. An outline of different subgroups of CAFs found in human pancreatic cancer, PDCA mouse model, lung and oral cancer

In human pancreatic cancer tissues, Ohlund et al. have found two major CAFs, one called myofibroblastic CAF (myCAF), which is located close to cancer cells and expresses high levels of α-SMA and FAP, has high levels of ECM-related genes, and is enriched in the TGF driver pathway. The other type of CAF is called inflammatory CAF (iCAF), which is distant from cancer cells and expresses low levels of α-SMA and FAP but secretes high levels of inflammatory mediators, including IL-6. By secreting cytokines and chemokines, iCAFs have more of a tumor-improving effect than myCAFs, indicating a more malignant biological behavior of pancreatic cancer. In contrast, myCAFs extensively deposit ECM to hinder drug delivery. The two can interconvert through paracrine factors [39, 40]. Grout et al. examined lung tumors via single-cell RNA sequencing combined with multiple imaging techniques and identified two CAF populations related to T cell marginalization, namely (i) myosin heavy chain 11 (MYH11)^+^SMA^+^ CAFs, which are present in early-stage tumors and form around aggregated cancer cells in a single cell layer; and (ii) FAP^+^SMA^+^ CAFs, which are present in more advanced tumors and are distributed in sheets in the stroma or in several layers around tumor clusters. Targeting of these CAF subpopulations may increase immunotherapy efficacy for tumors with marginalized T cells [41]. CAFs in oral cancer can be classified into C1 and C2 types by a low or high expression of α-SMA. C1-type CAFs regulate the proliferation and stemness of oral cancer cells; they are more supportive of cell proliferation but are not conducive to the self-renewal growth of stem-like cancer cells. C2-type CAFs are associated with the invasion and metastasis of oral cancer [42]. The above CAFs subgroups are showed in Fig. 2.

α-SMA^+^ CAFs are associated with worse prognosis in oral tongue squamous cell carcinoma (OTSCC) [43], head and neck squamous cell carcinoma (HNSCC), EAC, colon cancer [44], prostate cancer [45], breast cancer [37], and bile duct cancer [36, 46]. The findings of α-SMA and pancreatic prognostic correlation are inconsistent. Fujita et al. found that patients with high SMA expression have a shorter survival [35], whereas Maehira and Yuzawa concluded that SMA^+^ CAFs are not associated with pancreatic cancer prognosis [47, 48].

In terms of treatment, the TGF-β and IL-1/JAK/STAT signaling pathways are related to the formation and interconversion of these two CAF subtypes. Patients can benefit from the combination of JAK inhibitors and TGFBR inhibitors.

#### Periostin

Periostin (POSTN) was originally isolated from a mouse osteoblast cell line as osteoblast-specific factor 2 and belongs to the matricellular protein family. In humans, POSTN expression can be increased by interleukins and TGF-β [49, 50]. The POSTN molecule comprises four fascia I domains, a cysteine-rich domain in the N-terminal region, and an alternative splicing domain in the C-terminal region [51–56]. POSTN binds to both type I collagen and fibronectin [56, 57], is involved in collagen fibrosis, and is also related to Th2-dependent immune responses and inflammation [58]. POSTN^+^ CAFs are mainly derived from NFs [59].

POSTN is overexpressed in a variety of cancer CAFs and promotes various biological processes, including invasion and metastasis, by binding to appropriate integrin receptors or affecting the microenvironment [60–62]. In EAC and ovarian cancer, CAF-derived POSTN acts as a ligand for integrins, activates the PI3K/Akt pathway, and induces the EMT, providing the impetus for cancer cell migration and invasion [63, 64]. CAFs promote lymph node metastasis by activating the integrin FAK/Src-VE-cadherin signaling pathway in lymphoendothelial cells and damaging the lymphatic endothelial barrier in cervical squamous carcinoma [65]. In addition to binding to integrins, POSTN can also affect the TME to promote tumor metastasis. Single-cell sequencing of gastric cancer tissues revealed that POSTN-expressing CAFs promote gastric cancer invasion and metastasis by degrading the ECM and attracting tumor-associated M2-like macrophages to form a niche conducive to metastasis [66]. In salivary gland adenoid cystic carcinoma, POSTN is a potential biomarker for EV-induced anterior niches in CAFs in lung metastasis [67].

POSTN^+^ CAFs are involved in maintaining tumor cell stemness. POSTN^+^ CAF-derived POSTN promotes the cancer stem cell (CSC) phenotype of HNSCC by activating protein tyrosine kinase 7 (PTK7)-Wnt/β-Catenin signaling through binding to PTK7 in cancer cells [68]. Evidence suggests that POSTN is a major part of the CSC niche that maintains stemness and the metastatic colonization of CRC [69].

POSTN^+^ CAFs participate in drug resistance. In melanoma, BRAF inhibitors activate B-linked proteins in CAFs, stimulating the secretion of POSTN, which activates PI3K/AKT signaling and then reactivates the ERK pathway inhibited by BRAFi/Meki, promoting drug resistance [70]. POSTN^+^ CAFs are associated with epithelial ovarian cancer primary chemoresistance and predict shortened progression-free survival after first-line chemotherapy [71].

POSTN overexpression in CAFs is associated with many adverse clinicopathological factors, such as higher T and clinical stages and a larger tumor volume in NSCLC [72]; highly cell-rich and macrophage-infiltrated pancreatic cancer [73]; and lymph node metastasis and distant metastasis in pancreatic cancer and CRC [69, 73]. POSTN^+^ CAFs are also related to poor prognosis in PDAC [73], CRC [69], NSCLC [72], EAC [64], gastric cancer [66], and ovarian cancer [63].

In terms of treatment, neutralization of POSTN with appropriate antibodies has been shown to reduce breast cancer metastasis to the lung [74, 75]. In addition, a DNA inducer that binds to POSTN has been shown to inhibit the growth and metastasis of breast cancer and may be used as a therapeutic tool for breast cancers with POSTN^high^ CAFs [76]. Inhibition of POSTN may also weaken resistance to chemotherapeutic agents in breast cancer [77].

#### Platelet-derived growth factor receptor

Platelet-derived growth factor receptor (PDGFR) is a tyrosine kinase receptor that is located on the surface of fibroblasts, neural precursor cells, astrocytes, and pericytes. There are two types: PDGFRα and PDGFRβ. By binding to PDGF family members, it initiates a coagulation-forming response, stimulates angiogenesis, and promotes tumor growth and metastasis [78, 79]. PDGFRα/β^+^ CAFs are mainly derived from NFs and, to a lesser extent, from pericytes, and,vascular smooth muscle cells [6].

PDGFRα/β^+^ CAFs support malignant biological behaviors of tumors. PDGFRβ and integrin A11 (ITGA11) strongly colocalize and lead to more aggressive breast cancer cells by activating JNK signaling [78]. PDGFR interacts with integrin α5β1 to promote cell contraction and thus organize the ECM, leading to the directional migration of prostate cancer cells [80]. PDGFRβ in CAFs can interact with PDGF-BB expressed by cervical cancer cells and promote cancer cell growth by upregulating the expression of heparin-binding epidermal growth factor (HB-EGF) and activating the EGFR signaling pathway [81]. In CRC, PDGFRβ-labeled CAFs increase the metastatic and invasive ability of cancer cells in a secreted glycoprotein stanniocalcin-1-dependent manner [82]. PDGFRβ is a novel marker of stromal activation in oral squamous cell carcinoma [83]. In a xenograft mouse model of lung cancer, PDGFRα has been shown to promote the recruitment of fibroblasts to the tumor infiltration front. PDGFRα/β^high^ CAFs have also been linked to lymph node metastasis and lymphovascular invasion in ovarian cancer [84] and pancreatic cancer [48] and lead to poorer prognosis in breast cancer [78], ovarian cancer [84], pancreatic cancer [48], and lobular breast tumors [85]. Inhibition of PDGFR signaling can restrain cervical cancer angiogenesis and cell proliferation [86]. In addition, a PDGFR inhibitor, dasatinib, partially reverses cancer-promoting CAFs to resting-state fibroblasts in lung adenocarcinoma (LUAC) and is a potential therapeutic strategy for LUAC [87].

#### Fibroblast-specific protein 1

Fibroblast-specific protein 1 (FSP-1) belongs to the S100 small calcium-binding protein family, also known as S100A4. Studies have demonstrated that FSP-1^+^ CAFs induce angiogenesis and promote cell motility and metastasis by producing ECM proteins and secreting cytokines, including Tenascin C (TNC), matrix metalloproteinases (MMPs), and vascular endothelial growth factor-A (VEGF-A) [88–90].

The main sources of FSP-1^+^ CAFs are NFs, vascular endothelial cells, epithelial cells, and adipocytes [33, 91–93].

FSP-1 is correlated with lymph node metastasis in cholangiocarcinoma [46] and breast cancer [94] patients. FSP-1 is positive in CAFs of primary breast cancer and in matched lymph node metastases but negative in fibroblasts of cancer-free lymph nodes. This suggests that the lymph node stroma imitates the microenvironment of primary breast cancer [94].

In addition to lymph node metastasis, FSP-1^+^ CAFs are also involved in lung metastasis. In HNSCC, knockdown of FSP-1 in CAFs reduces the invasion and lung metastasis of cancer cells in xenograft tumor animal models by decreasing MMP2 expression, secretion, and activity [95]. FSP-1 induces lung metastasis in rodent models of breast cancer by increasing cell motility and invasion, which is not dependent on ECM degradation [96]. In a transgenic mouse model, FSP-1^+/+^ fibroblasts induce a massive infiltration of T cells and release of specific cytokines in the pre-metastatic lung, providing a favorable microenvironment for metastasis formation and leading to increased lung metastasis [97].

Because FSP-1^+^ CAFs are involved in lymph node metastasis and lung metastasis, elevated levels of FSP-1 in CAFs have been related to poor survival in breast cancer, CRC, and intrahepatic cholangiocarcinoma patients [46, 98, 99].

FSP-1^+^ CAFs drive metastasis by affecting the TME, making them an appealing target for anticancer therapy. Anti-FSP-1 monoclonal antibody effectively reduces T cell infiltration in fibroblast monolayers, inhibits the invasive growth of mouse and human fibroblasts, and reduces the metastatic burden in the lungs of experimental animals. Therefore, we hypothesize that FSP-1 expressed in CAFs could be a target for anti-metastatic therapy [100].

#### Palladin

Palladin is an actin-binding protein that serves as a scaffold to connect actin bundles, stress fibers, focal adhesions, Z-discs, and subcellular structures that play a critical role in normal cell motility [101, 102]. Palladin^+^ CAFs are mainly derived from NFs and stellate cells [103, 104].

There are nine isoforms of palladin. Of these, isoforms 3 and 4 (ISO3/ISO4) are highly expressed in CAFs of quantities of malignancies, such as pancreatic, lung, colon, and gastric cancer. Palladin regulates the expression of various genes encoding ECM proteins, which functionally affect the behavior of CAFs, leading to a pro-invasive phenotype [105].

In PDAC, CAFs with high expression of ISO3/ISO4 can effectively secrete immunosuppressive cytokines (TGF-β1, IL-8, and IL-6) to maintain the characteristics of inflammatory CAFs and produce functional desmoplastic ECMs (d-ECMs) that support tumor cell survival and proliferation [106]. In addition, high expression of palladin in CAFs facilitates the invasion of cancer cells by remodeling the ECM through regulation of the small GTPase Cdc42, which is considered to be the main regulator of core actin polymerization in invasive pseudopodia [104].

In breast cancer, palladin is an Akt1-specific substrate [107]. Downregulation of palladin by miR-96/miR-182 in CAFs reduces breast cancer cell migration and invasion [108]. In CRC [109], PDAC [110], and renal cell carcinoma [111], palladin^high^ CAFs are correlated with reduced survival.

#### Twist

Twist1 is a basic helix-loop-helix transcription factor that recruits nucleosome remodeling deacetylase (NuRD) to modify target gene-bound histones and participate in epigenetic processes in cells [112]. The main source of Twist1^+^ CAFs is NFs [113].

Twist1 expressed in CAFs is a marker of tumor progression. Twist1 expression in NFs can transdifferentiate them into CAFs via STAT3 phosphorylation and is sufficient and necessary for CAF transdifferentiation [113]. Twist1 is more commonly expressed in gastric CAFs than in those of other cancer species and is rarely expressed in non-cancerous tissues [114]. Increased expression of Twist1 in gastric cancer CAFs contributes to gastric cancer progression and worse patient survival while its increased expression in NFs can drive CAF marker expression and invasive features of gastric cancer cells both in vivo and in vitro. Inversely, silencing of Twist1 expression in CAFs abolishes their tumor-promoting characteristics [113]. Twist1 expression in CAFs is related to tumor size, lymph node metastasis, and depth of invasion, as well as poor survival in patients with gastric cancer, especially in patients with diffuse-type gastric cancer. Besides gastric cancer, Twist can be expressed in both cancer cells and CAFs of invasive ductal breast carcinoma and both are related to shorter patient survival, indicating Twist as a potential useful prognostic marker in invasive ductal breast carcinoma [115]. In CRC, Twist1 expression is mostly limited to the tumor stroma. It improves matrix stiffness by upregulating collagen α1 and palladin and promotes cell migration and invasion [109].

#### Glutamine-fructose-6-phosphate transaminase 2

Glutamine-fructose-6-phosphate transaminase 2 (GFPT2) is the rate-limiting enzyme of the hexosamine biosynthesis pathway (HBP), responsible for glycosylation [116]. Activation of HBP leads to altered O-GlcNAcylation and N-/O-glycosylation of transcription factors and kinases in various types of cancer. This process will stabilize proteins involved in tumorigenic processes, such as c-Myc and β-catenin, leading to proliferation, invasion, metastasis and drug resistance of tumor cells [117–122]. The main source of GFPT2^+^ CAFs is NFs [116].

In LUAC, the GFPT2 gene is upregulated after transformation of NFs into CAF-like cells by TGF-β, leading to increased glucose uptake. GFPT2 is predominantly expressed in invasive-edge CAFs and is a key regulator of tumor metabolic reprogramming. Its high expression in CAFs is negatively correlated with LUAC patient prognosis [116].

In CRC, GFPT2 is highly expressed, and CAFs are the main cells expressing GFPT2. GFPT2^+^CAFs positively correlates with immunosuppressive cells and T-cell exhaustion, implying that GFPT2 may be involved in immune escape of tumor cells. It regulates immunosuppression mainly through the JAK/STAT signaling pathway. Its high expression in CAFs is significantly associated with poorer pathological characteristics and OS in patients with CRC [123].

In addition to being expressed in CAFs, GFPT2 is also expressed in tumor parenchymal cells. The high expression of GFPT2 is closely related to invasion, metastasis, drug sensitivity, and poor prognosis of a variety of tumors including lung cancer [124, 125], epithelial ovarian cancer [126–128], CRC [129], breast cancer [130, 131], GC [132] and leiomyosarcoma [133].

### Markers of CAFs derived from MSCs

#### Vimentin

Vimentin is a type III intermediate filament protein that acts as a cytoskeletal protein and is localized to the cytoplasm. It is an EMT biomarker that maintains cell structure and motility during cell migration. In the TME, it is expressed not only in CAFs, but also in epithelial cells undergoing EMT, vascular endothelial cells, and mesenchymal-derived cells such as adipocytes and myocytes [134]. Vimentin^+^ CAFs are mainly derived from MSCs [34], although other sources include vascular endothelial cells and epithelial cells [6].

Vimentin^+^ CAFs are related to tumorigenesis, metastasis, recurrence, drug resistance and poor prognosis in patients of several cancers.

In CRC, vimentin expression in CAFs may reflect the higher malignant potential of the tumor. Vimentin^+^CAFs are significantly associated with a higher rate of disease recurrence, regardless of lymph node status, and is therefore an independent prognostic factor for CRC recurrence. High expression of vimentin in CAFs is also associated with shorter survival in patient with CRC [135]. Another study on CRC has found that stromal vimentin expression is significantly correlated with T stage, suggesting its possible involvement in tumor invasion. Stromal vimentin expression is an independent prognostic factor for CRC-specific survival (CSS) and disease-free survival (DFS) of high-risk stage II patients. Moreover, high-risk stage II patients with how stromal vimentin expression benefit less from standard adjuvant chemotherapy than those with low stromal vimentin expression [136].

In PDAC, Maehira H et al. used double immunofluorescence staining to divide the vimentin-positive CAFs into two subgroups: α-SMA co-expression and α-SMA no co-expression. Vimentin^+^ CAFs without co-expression of α-SMA are an independent predictor of poor survival in PDAC patients [47]. Through molecular and functional analysis of primary cultures of CAF derived from PDAC patients, Neuzillet C et al. have found that subtype A CAF cultures displayed low expression of vimentin and α-SMA, and may be associated with a less pro-tumoural (less pro-proliferative and chemoprotective to cancer cells) profile than other non-subtype A CAFs [137].

In gastric cancer, strong immunoreactivity of vimentin is detected only in stroma cells, most of which are CAFs [138, 139]. Vimentin^+^ CAFs are mainly related to higher T stage, Lauren classification(diffuse type), and higher serum carcinoembryonic antigen level [138, 139]. They can promote EMT in cancer cells by secreting elastin fibers, leading to distant metastasis. Therefore, vimentin^+^CAFs are an independent prognostic factor, and associated with recurrence, distant metastasis and reduced survival in patients with gastric cancer [138, 139].

During the progression of esophageal cancer initiated by chronic inflammation, vimentin^+^ α-SMA^+^ myofibroblasts migrate to sites of intestinal metaplasia and dysplasia, and may become an innocent bystander in tumor progression by directly enhancing proliferation of nearby epithelial cells in an NF-κB-dependent manner as well as indirectly by recruiting and polarizing cells of the adaptive and innate immune system toward a tumor-promoting phenotype [140].

In breast cancer, vimentin is conveniently abundant in connective tissue septa, especialy CAFs, but usually absent from the tumour parenchyma. It is associated with indices of poor prognosis, such as high growth fraction/S-phase, lack of oestrogen receptor, and poor nuclear grade [141].

In a genetic engineering mouse model of lung cancer, vimentin^+^ CAFs have been found to surround the cancer cells sprouting from the primary tumor to improve lung cancer metastasis [142]. As a result, vimentin may be used as a target for anti-metastasis therapy [142].

In oropharyngeal squamous cell carcinoma (OPSCC), the vimentin expression in CAFs is stronger among HPV-positive tumors than HPV-negative tumors. That suggest vimentin^+^ CAFs may be involved in the development of OPSCC due to HPV infection [143].

#### Tenascin C

TNC is an ECM protein that assembles into a hexamer via a disulfide bond at the N terminus. TNC comprises several functional structural domains, including a C-terminal globular structural domain, a heptapeptide repeat sequence, an epidermal growth factor-like repeat sequence, and a fibronectin type III structural domain. The latter domain can combine with other cell surface receptors and ECM molecules such as integrins and fibronectin [144]. TNC^+^ CAFs are mainly derived from MSCs [34].

In pancreatic cancer, high expression of TNC in CAFs activates downstream signaling through the Annexin II receptor, promotes EMT, and leads to distant metastasis [145, 146]. In addition, TNC^+^ CAFs are significantly related to lymph node metastasis in breast cancer [147] and prostate cancer [45] and associated with an advanced clinical stage and an elevated microvessel density and tumor-associated macrophages (TAMs) population in prostate cancer [45].

Based on the ability of TNC to promote tumor development, the high expression of TNC in CAFs is an independent prognostic marker for multiple cancers, including breast cancer [147], bladder cancer [148], prostate cancer [45], CRC (especially stage II and III CRC) [149, 150], and pancreatic cancer [151]. TNC combined with other TME components, such as low stromal caveolin-1 and CD8^+^ T cell number, could be used as significant prognostic markers in patients with NSCLC [152].

#### CD90

CD90, encoded by THY1, is a glycosylated phosphatidylinositol-anchored glycoprotein expressed on a variety of cells, including activated microvascular endothelial cells, blood stem cells, neurons, and fibroblasts [153–155]. CD90 is a notable regulator of cell–matrix and cell–cell interactions and plays a major role in neural regeneration, cell migration, adhesion, and fibrosis [156]. The main source of CD90^+^ CAFs is MSCs [34] while stellate cells are another source [157].

CD90^+^ CAFs promote the malignant biological behavior of tumors. They are the primary source of IL-6, which maintains the stemness of CRC cells and induces an adaptive immune inflammatory response favoring tumor growth [158]. CD90^+^ CAFs promotes tumor cell invasion by enhancing tumor cell–endothelial cell attachment in a KrasG12D-driven LUAC (KrasLA1) mouse model [159]. In hepatocellular carcinoma (HCC), a significant number of CAFs highly express CD90, which correlates with angiogenesis markers (CD105, CD34, and CD31) and is related to poor prognosis in HCC patients [160]. As a marker of activated stellate cells in pancreatic cancer, CD90^+^ CAFs may participate in the tumor–stromal interaction and promote cancer progression [157]. It could serve as a prostate cancer biomarker for CD90^+^ CAFs and distinguish prostate cancer-related stroma from benign stroma [161].

#### CD10 and GPR77

CD10, also known as common acute lymphoblastic leukemia antigen (CALLA), is a 90–110-kDa protein belonging to the zinc-dependent type II metalloproteinase family, an endopeptidase that degrades various bioactive peptides in the ECM [162]. Bone marrow-derived MSCs are the main source of CD10^+^ CAFs [163]. In addition, pre-B lymphocytes can also differentiate into CD10^+^ CAFs [6].

In breast cancer, CD10^+^ CAFs are related to estrogen receptor (ER)-negative invasive breast cancer, whereas CD10^−^ CAFs are associated with luminal-type invasive breast cancer [162].

In UC, CD10 expression in CAFs is significantly associated with poor prognostic clinicopathological factors, such as squamous differentiation, lymph node metastasis, and tumor necrosis. CD10 expression in cancer cells and CAFs are both related to a high tumor grade and shorter overall survival [164].

GPR77 belongs to the non-G protein-coupled receptor family [165]. Bone marrow-derived MSCs and polymorphonuclear neutrophils [6] are the main sources of GPR77^+^ CAFs. It has been found that CD10 and GPR77 can define human CAF subpopulations, and CAF subsets with high expression of CD10 and GPR77 are related to chemotherapy resistance and poor prognosis in patients with lung and breast cancer. CD10^+^GPR77^+^ CAFs supply consistent paracrine IL-8 and IL-6 through continuous nuclear factor-kappa B (NF-κB) signal transduction maintained by p65 acetylation and phosphorylation, forming a niche that protects CSCs from chemotherapy-induced cell death [163].

In a breast cancer patient-derived xenograft model, blockade of GPR77 with neutralizing monoclonal antibody markedly reduced the infiltration of CD10^+^GPR77^+^ CAFs and the proportion of ALDH1^+^ CSCs, resulting in decreased tumorigenesis and increased chemosensitivity [163].

### Markers of CAFs derived from stellate cells

#### Galectin 1

Galectin 1 (Gal1) is a β-galactoside-binding protein that bidirectionally regulates cell proliferation in a cell-specific and dose-dependent manner. It improves angiogenesis via the vascular endothelial growth factor receptor 2 (VEGFR2) pathway and participates in immune regulation by inducing T cell apoptosis and suppressing T cell proliferation and antigen-presenting cell activation [166]. Stellate cells can differentiate into Gal1^+^ CAFs with TGF-β induction [167].

In breast cancer, CAF-derived Gal1 helps to delimitate the immunosuppressive microenvironment by shifting it to a Th2 cytokine profile and increasing the frequency of Treg cells [168, 169]. Gal1^+^ CAFs participate in different events in PDAC, including tumor cell proliferation, angiogenesis, invasion, metastasis, and inflammation [103, 167]. Stromal Gal-1 promotes the cancer-initiating cell trait and disease dissemination in CRC via β-catenin and SOX9 [170]. High Gal-1 expression in CAFs is related to a worse clinical outcome in lung and breast cancer patients [168, 169, 171].

### Markers of CAFs derived from epithelial cells

#### Adipocyte enhancer-binding protein 1

Adipocyte enhancer-binding protein 1 (AEBP1) is a widely expressed multifunctional protein, especially in preadipocytes and macrophages, that produces a variety of inflammatory mediators, including tumor necrosis factor α (TNFα), monocyte chemotactic protein 1 (MCP-1), and IL-6 [172, 173]. AEBP1 has been found to be intimately related to EMT, suggesting that epithelial cells may be the main source of AEBP1^+^ CAFs [173].

Recent studies suggest that AEBP1 in the stroma may play a major role in cancer promotion. AEBP1 is related to numerous human malignancies, including gastric cancer, colon cancer, glioblastoma, and melanoma [174].

AEBP1 overexpression was recently found in CRC CAFs. It promotes proliferation, invasion, migration, and metastasis by activating NF-κB signaling. In melanoma and CRC, high expression of AEBP1 in CAFs is positively correlated with both fibroblast biomarker expression and EMT meta scores, suggesting that AEBP1 is responsible for EMT and that AEBP1-mediated EMT may promote fibroblast activity [174, 175]. AEBP1 is upregulated in CAFs of lung squamous carcinoma patients and is an independent prognostic factor in both univariate and multivariate analyses [176].

### Cancer-promoting CAF markers of unclear origin

#### Osteopontin

Osteopontin (OPN) is a secretory phosphoprotein. As a cell attachment protein and cytokine, it signals through two kinds of cell adhesion molecules—integrin V-3 and CD44—to participate in tumorigenesis [177–180]. The source of OPN^+^ CAFs is not clear at present.

CAF-secreted OPN promotes cancer stemness via the secreted phosphoprotein 1(SPP1)–CD44 axis in pancreatic cancer [181]. It combines with avb3 and CD44, which activates the STAT3/PPARγ pathway and promotes macrophage M2 polarization and finally contributes to CRC tumorigenesis [182]. In lung cancer, OPN expressed by CAFs acts as a potential biomarker of invasiveness and proliferation [183]. Anti-OPN monoclonal antibody (AOM1) alone or coupled with carboplatin markedly suppresses the growth of lung metastases, thereby demonstrating the important function of OPN in tumor metastasis and progression [184]. The expression of OPN is related to poor survival in patients of various tumor types such as breast, lung, and colorectal cancers [185–188].

## CAF markers with cancer-restraining function

### Meflin

Meflin is a glycosylphosphatidylinositol-anchored protein encoded by the ISLR (immunoglobulin superfamily leucine-rich repeat sequence) gene [189, 190]. It can also be secreted into the culture medium of fibroblasts and specifically expressed in CAFs of human and mouse PDAC [190–193].

Meflin expression in CAFs of early pancreatic cancer effectively attenuates tumor aggressiveness. Patients with high levels of meflin-expressing CAFs are more likely to have highly differentiated pathological histological features and better prognosis, and meflin deficiency in CAFs can lead to more aggressive behavior and resistance to chemotherapeutic agents in PDAC. This suggests that meflin is a functional marker of rCAFs in PDAC [192, 194].

Meflin^+^ CAFs are associated with a favorable therapeutic response to immune checkpoint blockade in patients with NSCLC. In NSCLC patients and mouse syngeneic tumor models, large numbers of meflin^+^ CAFs attracted greater CD4^+^ T cell infiltration and promoted tumor angiogenesis, which in turn enhanced the response of NSCLC to immune checkpoint blockade therapy [195].

Interestingly, meflin^+^ fibroblasts are found in the mesenchyme of non-neoplastic fibrotic diseases, such as cardiac fibrosis, idiopathic pulmonary fibrosis, and renal fibrosis. They play an essential role in tissue repair and in inhibiting fibrosis [196–198]. To date, two proteins have been identified to interact with meflin. One is bone morphogenetic protein 7 (BMP7), a cytokine to which meflin binds, activating downstream Smad1/5 and functionally counteracting the profibrotic function of TGF-β [196, 199, 200]. Another meflin ligand is lysyl oxidase (Lox), a cross-linking agent of collagen fibers that promotes fibrosis and tissue sclerosis [194, 201–203]. Meflin interacts with Lox and inhibits its collagen cross-linking agent activity, which in turn inhibits fibrosis formation [194].

Meflin has been found to act as a marker of reversed CAFs with elevated drug sensitivity. A lineage tracing experiment has shown that meflin^+^ rCAFs differentiate into α-SMA^+^ meflin^−^ CAFs, known as pCAFs, in the course of cancer progression. AM80, a synthetic unnatural retinoid, is an effective agent for the conversion of meflin^−^ pCAFs to meflin^+^ rCAFs, enhancing drug distribution and chemosensitivity via the conversion of CAFs [204].

### CD146

CD146 is a cell adhesion molecule originally considered a melanoma-specific cell adhesion molecule [205]. It participates in the immune response, MSC differentiation, cell development, cell migration, signal transduction, and angiogenesis [206]. The main source of CD146^+^ CAFs is vascular endothelial cells [6].

In ER^+^ breast cancers, CD146 distinguishes two CAF subgroups: CD146^+^ CAFs maintain ER expression, estrogen-dependent proliferation, and sensitivity to tamoxifen, whereas CD146^−^ CAFs play the opposite role [207]. The expression of CD146 is higher in PDAC CAFs than in cancer cells, and CD146 expression in CAFs is related to low-histological grade PDAC and high-grade pancreatic intraepithelial neoplasia. Patients with CD146^low^ CAFs have a worse prognosis. In vitro experiments using primary cultures of CAFs isolated from PDAC tissues have shown that blockade of CD146 expression in CAFs significantly increases the invasion and migration of cancer cells. Furthermore, knockdown of CD146 promotes CAF activation by inhibiting the NF-κB pathway and upregulating the secretion of CAF chemokines and cytokines, such as hepatocyte growth factor (HGF), CXCL1, SDF1A, COX2, and CCL5. In conclusion, the decreased expression of CD146 in CAFs promotes pancreatic cancer progression [208].

## CAF markers with bidirectional functions

### Caveolin-1

Caveolin-1 (Cav-1) is a scaffolding protein and a major component of cell surface depressions [209]. It participates in numerous physiological functions, such as intracellular cholesterol transport, endocytosis, and cell surface signaling [210–214], and plays a significant role in cellular senescence [215, 216]. Cav-1^+^ CAFs can be derived from NFs, vascular endothelial cells, and adipocytes [6].

In cancer, Cav-1 mainly participates in various processes, including cell transformation, tumor growth, invasion and metastasis, multidrug resistance, and angiogenesis [217, 218]. Downregulation of Cav-1 in the TME promotes the transformation of adjacent NFs into CAFs [219]. Promoter hypermethylation is the main cause of the silencing of Cav-1 gene expression [220].

Cav-1 in CAFs has been found to have tumor-suppressive properties, and deletion of Cav-1 predicts poor prognosis. In breast cancer, Cav-1 deletion from CAFs is related to a more advanced tumor stage, early cancer recurrence, lymph node metastasis, and poor prognosis. In patients selected for triamcinolone therapy, Cav-1 deletion in CAFs is a strong predictor of a poor clinical prognosis and can indicate triamcinolone resistance [217, 221–223]. In addition to the findings from breast cancer, Cav-1^low^ CAFs in prostate and gastric cancers also predict a poor prognosis [217, 223]. In a phase II clinical trial of NSCLC patients treated with nab-paclitaxel, Cav-1 expression in CAFs was related to prolonged survival and increased response rates, indicating that Cav-1 is a marker of nab-paclitaxel treatment efficacy [224].

The tumor-suppressive properties of Cav-1 expressed in CAFs can be achieved via several mechanisms. First, Cav-1 deficiency can cause EMT. Pavlides et al. have found that CAFs with Cav-1 deficiency overexpress multiple growth factors, such as PDGF, VEGF, TGF-β, and IL-6, and stimulate normal mammary epithelial cells to generate EMT in a paracrine manner [209]. Second, Cav-1 deficiency also promotes the malignant phenotype of tumors by affecting tumor metabolism. Several studies have shown that, based on the reverse Warburg effect theory, the upregulation of TGF-β caused by Cav-1 deletion can induce fibroblasts to undergo aerobic glycolysis and produce lactic acid while promoting the transformation of fibroblasts to myofibroblasts, which exhibit more pronounced activity in oxygenated glycolysis and autophagy, providing energy material to neighboring cancer cells. This metabolic symbiosis also contributes to the malignant phenotype of the cancer, and the activation of autophagy in the tumor stroma leads to chemoresistance [225, 226]. Third, a progressive reduction in Cav-1 expression is involved in radioresistance in prostate cancer through upregulation of TRIAP1 (TP53-regulated inhibitor of apoptosis 1) [227].

However, some studies have come to the opposite conclusion, suggesting that Cav-1 expression in CAFs is a potential indicator of cancer progression [33, 228, 229]. Goetz JG et al. have found that Cav-1 expression is significantly higher in breast cancer CAFs than in NFs and that the 10-year risk of death is 2.5-fold higher in patients with Cav-1^high^ than in controls, suggesting that Cav-1 in CAFs has the potential to improve tumor cell invasion and metastasis by regulating Rho-mediated cell contractility in a p190-dependent manner, remodeling the tumor stroma and intra-tumor microenvironment and hardening the TME [228]. In pancreatic cancer, the CA19-9 level and rates of an advanced tumor stage are markedly higher in CAV1^high^ patients than in controls. Moreover, the disease-free survival and overall survival are markedly lower in patients with high CAV1 than in controls. The same conclusion was also reached for renal cell carcinoma, prostate cancer, colon cancer, and melanoma [33, 228, 229].

The above results indicate that Cav-1^+^ CAFs play a distinct role in tumor progression. Treatments targeting Cav-1 may require a completely different strategy.

### Podoplanin

The mucous sialoglycoprotein podoplanin (PDPN) is widely used as a histopathological marker to differentiate lymphatic vessels from blood vessels due to its expression on lymphatic vessel endothelial cells [230, 231]. However, recent studies have reported that PDPN is also expressed in cancer cells, dendritic cells, and inflammatory macrophages, especially CAFs [232–234]. A previous study showed that PDPN is present in extracellular and intracellular regions [235]. The binding of its intracellular regions to Ezrin, Radioxin, and Moesin (ERM) proteins is thought to lead to cell morphological changes and cytoskeleton reorganization [236]. PDPN^+^ CAFs are mainly derived from vascular endothelial cells [6].

PDPN^+^ CAFs exert tumor-promoting functions by facilitating the invasion of cancer cells [237], remodeling of the ECM [234, 238, 239], and promoting an immunosuppressive microenvironment [240].

In LUAC, the CD204^+^ TAM population is significantly larger and the CD8/FOXP3 T cell ratio markedly lower in PDPN^+^ CAF patients than in PDPN^−^ CAF patients, indicating that PDPN induces the polarization of M2 macrophages and suppresses the gene expression levels of cytokines of immune-related lymphocytes and thereby allowing LUAC to be characterized by an immunosuppressive microenvironment [240]. In stage I lung squamous cell cancer, PDPN^+^ CAFs highly express TGF-β1 and are associated with the infiltration of CD204^+^ TAMs, further demonstrating that PDPN^+^ CAFs can lead to an immunosuppressive microenvironment [241]. In addition, PDPN^+^ CAFs induce primary resistance to EGFR tyrosine kinase inhibitor (EGFR-TKI) in LUAC with EGFR mutation by activating the MAPK signaling pathway in cancer cells [242].

PDPN^+^ CAFs are associated with various adverse clinicopathological factors and poor prognosis. They are related to lymphatic vessel invasion and/or lymph node metastasis in EAC [243], perihilar cholangiocarcinoma [244], PDAC [245, 246], and melanoma [247]; an advanced tumor stage, including TNM stage in perihilar cholangiocarcinoma [244] and T stage in EAC [244] and PDAC [245, 246]; a high histological grade in PDAC [245, 246]; and a shorter survival time in EAC [243], PDAC [245, 246], melanoma [247], lung squamous cell cancer [248], and LUAC [240].

However, in breast and colorectal cancers, the opposite findings have been obtained. In invasive breast cancer, PDPN-expressing CAFs are a favorable independent prognostic factor [249]. In CRC, high PNPD expression in CAFs is protective against CRC cell invasion and is a major sign of good prognosis in patients with advanced CRC [250]. Another study has found that PDPN^+^CAFs are abundantly present in the tumor center rather than the infiltrative margins, and they are associated with favorable clinicopathological parameters and prolonged tumor-free survival [251]. As a mucin-type glycoproteins, PDPN has an extended brush-like conformation due to their extensive O-glycosylation [252]. This highly negatively charged structure is relatively resistant to proteases and provides a physical barrier protecting cells from environmental agents. In addition, the PDPN^+^ CAFs locate mainly in the superficial to deep area of the tumor, sparing the invasive front where tumor budding is often observed. The above reasons support the idea that podoplanin may play an important protective role against cancer invasion [215, 216].

The above results suggest that cancer cells respond differently to PDPN^+^ CAFs in different cancers or that the distinct functions of PDPN^+^ CAF in various organs may account for the different roles of PDPN in different cancers.

### CD200

CD200 (OX2), an OX2 membrane glycoprotein, is normally expressed in hair follicle epithelial cells, neurons, lymphocytes, MSCs, and monocytes [253]. CD200 interacts with the CD200 receptor (CD200R1) to trigger an immunosuppressive response [254].

Recent studies have shown that CD200 can be expressed in CAFs. Analysis of samples from LUAC patients with an EGFR gene mutation treated with the EGFR-TKI gefitinib after surgery showed that those with CD200^+^ CAFs in resected LUAC tended to have longer progression-free survival in terms of postoperative recurrence. Further studies showed that a subpopulation of CD200^+^ CAFs exhibited increased gefitinib sensitivity due to an enhanced pro-apoptotic effect of gefitinib on cancer cells and that downregulation of CD200 expression deprived CAFs of their sensitizing ability. The above results indicated that CD200^+^ CAFs may play a major role in the therapeutic application of EGFR-TKIs [255].

In pancreatic cancer, CD200 is positively expressed in CAFs and negatively expressed in cancer cells, but no clear correlation is seen with either progression-free survival or overall survival [256]. However, as a regulator of myeloid cell activity, CD200 expression in PDAC CAFs limits the response of cancer cells to PD-1 immune checkpoint inhibitors by enhancing myeloid-derived suppressor cell activity. Blockage of CD200 significantly improves the effect of immunotherapy [257].

Samalizumab, a CD200 immune checkpoint inhibitor, showed favorable results in a phase I clinical trial of patients with chronic lymphocytic leukemia [258].

## Other CAF markers

Several other markers have been found to be highly expressed in CAFs. Microfibril-associated protein 5 (MFAP5) is highly expressed in CAFs of tongue squamous cell carcinoma and is associated with the activation of multiple pro-growth signaling pathways, such as MAPK signaling. This indicates that MFAP5 may play a role in the identification of key oncogenic CAF subpopulations [259]. A new class of CAFs expressing MHC class II and CD74 has the ability to deliver antigens to CD4^+^ T cells and may regulate the immune response of PDAC and has been named the “antigen-presenting CAFs” [260]. CD70, which is more abundantly expressed in regulatory T cells, is highly expressed in CAFs of CRC and is negatively correlated with survival [261]. In addition, two markers, Transgelin (TAGLN) [59] and collagen type XI alpha I chain (COL11A1) [262], are also considered to be highly specific CAF markers. Their use in research is still limited, and further studies are needed to clarify the behavioral characteristics of these CAF markers in different cancers.

## Summary

In summary, there are many CAF markers and, in addition to the traditional known markers, a variety of new markers have been discovered in recent years. However, there is still no clear marker to identify CAFs, and even the most commonly used FAP and α-SMA still have some unavoidable inaccuracies when used alone. First, the selection of markers is limited by cancer species, and even the same marker has different classification criteria among different cancer species. Second, multiple markers are required in combination to refine the specific functions of different CAF subtypes. The use of new methods, such as spatiotemporal single-cell transcriptome and proteome analyses and lineage tracing, will reveal a more exhaustive landscape of CAF differentiation and diversity in various cancers, allow us to better comprehend the behavior and function of CAFs and shed light on the crosstalk between genetic mutations in cancer cells and CAF heterogeneity. These approaches have implications for the identification of targets for tumor therapy. Third, we can reverse the pCAFs into rCAFs by targeting the appropriate CAF marker, which can compensate for the current deficiencies of cancer therapy by only targeting the cancer cells themselves. Finally, expansion of the definition of the TME by linking CAFs with other non-tumor cells in the TME, such as immune cells, can also make up for the deficiencies of CAFs themselves.

## Data Availability

All data included in this study are available upon request by contact with the corresponding author.
